# Navigating job satisfaction in family firms during crisis

**DOI:** 10.3389/fpsyg.2024.1285221

**Published:** 2024-02-13

**Authors:** Maria Jose Ibañez, Nelson A. Andrade-Valbuena, Orlando Llanos-Contreras

**Affiliations:** ^1^CENTRUM Católica Graduate Business School, Lima, Peru; ^2^Pontificia Universidad Católica del Perú, Lima, Peru; ^3^Faculty of Economic and Administrative Sciences, Universidad Católica de la Santísima Concepción, Concepción, Chile; ^4^Facultad de Economía y Gobierno, Universidad San Sebastián, Sede Concepción, Concepción, Region del Biobio, Chile

**Keywords:** family firms, job satisfaction, crisis, affective commitment, burnout, COVID-19

## Abstract

Occupational health is one of the aspects significantly affected during crisis periods. It is essential to learn about the factors that improve organizational capacity in coping with such shocks. This study investigates how the working environment of a family business influences job satisfaction during crises. Conducting a survey with 516 employees at the peak of the pandemic, the research utilizes structural equation analysis, revealing that family business environments can mitigate burnout, enhance affective commitment, and consequently, boost job satisfaction. The study highlights the need to manage burnout and utilize resources, such as employee commitment, for family firms to sustain job satisfaction amidst disruptions. It deepens the comprehension of family businesses’ crisis response, emphasizing the significance of human resource commitment and management. The investigation illuminates the dynamic interplay between the work environment, employee well-being, and organizational resilience, providing valuable insights for both theoretical understanding and practical application.

## 1 Introduction

Family businesses constitute a predominant organizational type, representing over 70% of enterprises globally and employing 50–75% of the worldwide working population (Poza and Daugherty, 2013). In Latin America, family firms make up 65–98% of private enterprises, with Chile exhibiting a particularly high dominance of family control at around 90% of all firms. These businesses enjoy elevated levels of legitimacy in the region, attributed to their survival-focused orientation, network-based relationships, and in-group solidarity (Berrone et al., 2022).

The advent of COVID-19 has presented new challenges for family business research, prompting investigation into whether these firms possess more resilient work environments in the face of external shocks. Existing research on family firms’ post-disaster recovery suggests such resilience (Danes et al., 2009; Mahto et al., 2022). Determining the elements supporting this resilience, particularly at the occupational health management level, becomes crucial (Calabro et al., 2021; Llanos-Contreras et al., 2023). This investigation is significant due to the substantial impact family firms have on economies and their social relevance as workplaces. Understanding this phenomenon is essential for these firms to effectively navigate external shocks while concurrently fortifying their working environments.

A means of evaluating occupational health is through job satisfaction (Giménez-Espert et al., 2020). Family firms exhibit both advantages and disadvantages in cultivating working environments that either enhance or preserve job satisfaction. On the downside, family firms tend to offer lower wages, fewer career development opportunities, limited decision-making authority, and diminished influence on work design and business activities for non-family employees (Block et al., 2018; Waterwall and Alipour, 2021). Conversely, they are recognized as positive stewards, providing high job security, fostering social relationships, cultivating strong organizational cohesion, and promoting shared values and vision (Arregle et al., 2007; Hauswald et al., 2016; Llanos-Contreras et al., 2022a).

A recent study suggests that the advantages outweigh the disadvantages in family firms’ working environments, leading to higher levels of job satisfaction despite lower wages for employees (Block et al., 2015). This aligns with the propositions of enhanced proactive stakeholder engagement and socioemotional wealth preservation (Cennamo et al., 2012; Mahto et al., 2022), indicating that family firms prioritize preserving social ties with stakeholders, yielding benefits in the quality of working environments (Llanos-Contreras et al., 2022a,b). This, in turn, creates a resources advantage in terms of employee identification and emotional commitment crucial for family firms to face external shocks and adapt to disruptive change (Hoekx et al., 2022; Mahto et al., 2022). Research also indicates that certain policies, such as those oriented towards job benefits like the Job Benefit of Care, are more effective in driving job satisfaction in family-owned firms (Querbach et al., 2022). While intriguing, it remains unclear whether these mechanisms (flow of process and policies) and structural organizational conditions (resources) yield the same results (employee satisfaction) when family firms confront external disruptions like the COVID-19 pandemic. To address this gap, this study aims to answer whether family business working environments exhibit higher levels of job satisfaction than non-family firms during an external shock like Covid-19 and how employees’ affective commitment (resource asymmetries) and their management of burnout (flow of process) influence job satisfaction.

To address these questions, this study employs Partial Least Squares Structural Equation Analysis (PLS-SEM) using survey data collected from 516 Chilean employees. The survey includes information on whether they work in a family firm, along with responses to assess their affective commitment, burnout, and job satisfaction. Implemented at the peak of the pandemic concerning sanitary restrictions and mortality rates, the results affirm that, amid the COVID-19 scenario, family business working environments exhibit lower levels of burnout and higher affective commitment, subsequently positively influencing job satisfaction. The findings also indicate that family business working environments demonstrate higher levels of job satisfaction when confronted with the challenges of the COVID-19 pandemic, but this effect is observed only when mediated by burnout and affective commitment. The direct effect was not confirmed. This suggests that a family firm’s capacity to preserve employees’ job satisfaction hinges on their ability to manage processes that control burnout during external shocks, coupled with resource advantages, such as heightened affective commitment, to navigate adverse scenarios like those imposed by the pandemic.

This study makes at least three contributions to the existing body of theory. Firstly, it adds to the literature examining how family firms navigated the COVID-19 crisis, extending beyond general management perspectives provided by previous research (Kraus et al., 2020; Llanos-Contreras et al., 2023). While prior studies focused on family firms’ reactions to disruptions caused by COVID-19, our research uniquely delves into human resources commitment and its management as a crucial factor influencing these firms’ resilience during sudden external disruptions, such as the pandemic. Secondly, the research contributes to the field of family business resilience (Danes et al., 2009; Salvato et al., 2020). Contrary to the assumption that merely being a family firm guarantees higher job satisfaction during external disruptions, our study highlights the necessity of factors like employees’ affective commitment and controlled burnout for achieving this outcome. Finally, the study contributes to the domain of family business human capital management research (Lambrechts and Gnan, 2022; Pelaez-Leon and Sanchez-Marin, 2023) by identifying and testing specific resources that confer advantages to these firms, such as heightened employee affective commitment. Additionally, it confirms the family firms’ ability to maintain lower levels of burnout compared to non-family firms when confronting external disruptions.

Following on, this article unfolds with a theoretical discussion supporting the hypotheses. The subsequent section details the research design and outlines the procedures followed for data collection and analysis. Following this, the results are presented and discussed, aligning with the existing literature that guided the study. The final sections encapsulate the main conclusions, theoretical contributions, and practical implications, while also addressing the study’s limitations and proposing avenues for further research.

## 2 Theoretical background

The COVID-19 pandemic has presented unparalleled challenges for organizations and their employees, introducing heightened stress, uncertainty, and work-life imbalance (Le Breton-Miller and Miller, 2022). Existing research indicates that the prioritization of preserving socioemotional wealth is crucial in elucidating family firms’ capacity to manage occupational health, specifically addressing burnout, and fostering employee commitment during challenging times. Socioemotional wealth serves as the primary motivator propelling these firms to persist despite minimal financial rewards (Glover and Reay, 2015). It also plays a pivotal role in explaining their continuity and survival in the face of natural disasters (Mahto et al., 2022). Moreover, socioemotional wealth is recognized as the driving force behind turnover following organizational decline (Llanos-Contreras and Jabri, 2019). In this context, socioemotional wealth emerges as a potential driving force compelling these firms to manage occupational health effectively, enhancing their resilience to survive external shocks such as the COVID-19 pandemic (Llanos-Contreras et al., 2023).

At the core of socioemotional wealth preservation lies these firms’ priority for fostering strong social ties with various stakeholders, with a particular emphasis on their staff (Cennamo et al., 2012; Llanos-Contreras et al., 2022b). Recently, Christensen-Salem et al. (2021) has indicated that the pursuit of socioemotional wealth explains why family firms exhibit a heightened concern for developing caring practices toward their employees. In a similar vein, Jennings et al. (2018) suggested that in family firms, employees are treated by owners as if they were part of the family. This may elucidate why non-family employees in family firms display higher levels of organizational commitment compared to employees in non-family firms (Pimentel et al., 2020). Furthermore, socioemotional wealth has been employed to clarify why family firms adopt a more cautious approach to firing practices during crises, resulting in enhanced social welfare and happiness within these firms (Rivo-López et al., 2022). This lends support to our proposition of an improved ability to manage burnout under crisis situations.

In the midst of the Covid-19 crisis, family businesses have demonstrated unique strategies utilizing resources and mechanisms to ensure their survival. Le Breton-Miller and Miller (2022) discovered that a long-term orientation, robust relationships with employees, and close connections with stakeholders empowered family firms to effectively navigate the challenges posed by the pandemic. Llanos-Contreras et al. (2023) highlighted how these firms strategically balanced business demands and resources to mitigate employees’ psychological risks amid the pressures of the Covid-19 pandemic. Alonso-Dos-Santos and Llanos-Contreras (2019), as well as Llanos-Contreras et al. (2020), argued that during crises, family firms place a heightened emphasis on fostering strong social ties with employees, considering it a cornerstone that bolsters the continuity of these businesses in the face of adversities. The literature on occupational health further supports the link between working conditions and the health and safety of employees in family business environments (Madden et al., 2020). Crucially, affective commitment, burnout, and job satisfaction emerge as key indicators shedding light on occupational health in the workplace (Amponsah-Tawiah and Mensah, 2016).

Drawing from the aforementioned research, it is suggested that family businesses cultivate more resilient working environments, characterized by a distinct organizational dynamic (process) that effectively manages psychological risks in the workplace. Simultaneously, these environments foster enhanced organizational commitment among employees and contribute to higher levels of job satisfaction. In this line, this paper elucidates the importance of the social environment in the development of individuals, highlighting the crucial role of environmental support in fostering personal wellbeing, and therefore, enhancement of organizational resilence (see Zhang et al., 2023).

### 2.1 Employees’ affective commitment and organizational ability to manage burnout in family firms under a pandemic scenario

Family firms exhibit distinctive characteristics that contribute to the creation of a unique organizational climate, particularly advantageous in managing psychosocial risks on workers’ health during external disruptions (Marshall and Schrank, 2020; Mahto et al., 2022; Llanos-Contreras et al., 2023). Their strong emphasis on family values, long-term orientation, and the cultivation of binding social ties create favorable working conditions for addressing external shocks (Miller and Le Breton-Miller, 2005). This environment proves beneficial in maintaining control over emotions, psychological risks, and employee satisfaction during disruptive events (Llanos-Contreras et al., 2019; Bartik et al., 2020). A key element contributing to this resilience advantage is the robust affective commitment of employees within these firms (O’Regan and Quigley, 1993; Tabor et al., 2018).

Affective commitment, defined as an employee’s emotional attachment, identification, and involvement with the organization, is marked by the joy of being a member of the organization (Meyer and Allen, 1991). In contrast to normative commitment and continuous commitment, affective commitment is distinguished by its emotional basis, reflecting a genuine attitude toward the firm (Boswell and Olson-Buchanan, 2004). Predictors of affective commitment include personal factors like seniority and education, as well as organizational factors such as organizational culture and leadership integrity (Li et al., 2022). Given family firms’ inclination toward a more familial environment compared to non-family firms, there is a heightened likelihood of promoting a sense of belonging and loyalty among employees (Tabor et al., 2018). Additionally, research by Rauscher et al. (2012) supports the notion that family firms offer a social context with protective effects for employees. The literature also underscores that family firms cultivate a supportive work environment, fostering strong relationships among employees (Bassanini et al., 2013; Christensen-Salem et al., 2021). Cumulatively, these findings suggest that family business working environments tend to develop strong levels of employee affective commitment. Consequently, we propose the following hypothesis:

*H1*: Workers’ perception of involvement in a family business enhances the sense of affective commitment of employees, when facing an external shock, such as Covid-19.

Burnout is characterized by emotional, physical, and mental exhaustion resulting from prolonged exposure to stressful work conditions, leading to heightened frustration, increased depersonalization, and diminished personal accomplishment (Maslach, 1998). Employee burnout is associated with discrepancies between job descriptions and actual conditions, work overload, risky work environments, and intense interactions with various stakeholders, including coworkers, superiors, and supervisors (Burke, 1989; Savicki and Cooley, 1994; Dormann and Zapf, 2004; Yagil, 2006; Swendiman et al., 2019). Consequently, burnout is closely tied to how firms manage their daily working activities (flow of process) and, in the case of sudden disruptive shocks, how these disruptions impact daily activities and the firm’s ability to cope with the traumatic situation (Prado-Gascó et al., 2020; Torrès et al., 2021).

Existing research suggests a correlation between environmental conditions in family firms and reduced levels of employee burnout. For example, Fotiadis et al. (2019) found evidence that family firms, given their emphasis on employee well-being and work-life balance, contribute to decreasing employee burnout. Additionally, family firms often provide more flexible work arrangements, such as telecommuting or adaptable scheduling, enabling employees to balance work and personal responsibilities, thereby alleviating anxiety and pressure that can lead to job burnout during crises (Stamm et al., 2022). The family-like work environment fostered by family firms may also enhance employees’ social adaptability and support, acting as a buffer against the negative effects of stress and burnout during crises (Schwaiger et al., 2022). These strengths collectively suggest a superior ability in family firms to manage employee burnout when confronted with external shocks such as COVID-19, supporting the following hypothesis:

*H2*: Workers’ perception of involvement in a family business decreases their burnout levels, when facing an external shock, such as Covid-19.

### 2.2 Employees’ affective commitment, burn-out and job satisfaction during the COVID-19 pandemic

Work-related stress is a common occurrence in the workplace, often leading to increased levels of burnout. Maintaining control over burnout levels is crucial, as moderate stress can introduce challenges and diversity to a job, contributing positively to the work environment. However, excessive stress poses the risk of detrimental outcomes (French et al., 2000). Since the seminal article from Maslach et al. (2001) hypothesized the negative relationship between burnout and job satisfaction, there has been a considerable amount of research providing empirical evidence on this in different contexts. One influencial study addressing health personnel is the meta-analysis of Zangaro and Soeken (2007). They provides wide evidence of the negative effects of burnout on job satisfaction. Similarly, the meta-analysis from Madigan and Kim (2021) supports that, in contexts of primary education, burnout is correlated to low levels of job satisfaction. In the same line, considering sales personnel, the meta-analysis of Edmondson et al. (2019), confirmed this relationship within the businesses context. These studies contribute to empirical research, offering substantial evidence confirming the negative relationship between the two constructs. For instance, Babakus et al. (1999) provide support for sales personnel within a large international service organization, based on a sample of 350 individuals. Wang et al. (2015) extend this evidence to Canadian teachers, with a sample size of 523. More recently, Ariawan et al. (2023) present additional support for employees in the electronic manufacturing sector in Indonesia. Notably, this last study is part of a broader set of investigations assessing this relationship during the COVID-19 pandemic (Ninaus et al., 2021; Lea et al., 2022; Anand et al., 2023; He et al., 2023; Kraus et al., 2023). All the aforementioned evidence supports the following hypothesis.

*H3*: Having lower levels of Burnout is related to higher levels of Job Satisfaction, when businesses face an external shock, such as Covid-19.

The concept of employees’ organizational commitment has been a focal point in organizational studies for a considerable period (Mathieu and Zajac, 1990; Lincoln and Kalleberg, 1992). It serves as a crucial factor influencing turnover and absenteeism and is recognized as a reliable predictor of a team’s capacity to navigate new challenges and organizational change (Porter et al., 1974; Febrianti and Jufri, 2022). Recent research by Chanana (2021) and Liu et al. (2021), focusing on teachers during the COVID-19 pandemic, identified a positive relationship between organizational commitment and job satisfaction. Affective commitment, a key element of organizational commitment, has been highlighted in studies such as Koo et al. (2020). Affective commitment pertains to an employee’s emotional attachment to their organization and their inclination to remain a part of it. It is rooted in recognition, emotional attachment, and active participation within the organization (Ko et al., 1997). Individuals exhibiting affective commitment remain devoted to the organization because they perceive that their personal employment relationship aligns with the goals, principles, and values of the organization (Beck and Wilson, 2000). Drawing on this understanding, it is suggested that in periods of external shocks, employees with elevated levels of affective commitment may experience a heightened sense of loyalty and dedication to their organization (Chanana, 2021). This would enhance employees’ resilience during external shocks, cultivating motivation and a commitment to continuity, ultimately preserving job satisfaction (Koo et al., 2020). This would be especially relevant in small and medium-sized family firms, where a significant number of employees are also family members (Cruz et al., 2012). Research on family businesses has revealed that bolstering cohesion and solidarity, factors related to affective commitment, is pivotal in elucidating the response mechanisms of family firms to the challenges posed by the COVID-19 crisis (Kraus et al., 2020). Consequently, within these firms, heightened affective commitment from employees appears to play a crucial role in sustaining job satisfaction when confronted with disruptive shocks. Therefore, the following hypothesis is posited.

*H4*: Having higher levels of affective commitment is related to higher levels of job satisfaction, when firms face an external shock, such as Covid-19.

### 2.3 Family firms and job satisfaction

The work environment in family firms can significantly influence employee job satisfaction, particularly during external shocks such as the COVID-19 pandemic (Lyu et al., 2021). Previous research on socioemotional wealth has demonstrated that family businesses prioritize non-financial goals, including fostering altruism within the family and the organization (Schulze et al., 2003) and building strong social ties with employees (Block, 2010). Studies indicate that in family firms, a positive work environment often arises from the strong ties and shared values within the organization (Herrero and Hughes, 2019), contributing to increased job satisfaction.

Several factors support the idea that family firms cultivate positive work environments conducive to job satisfaction. Family members, who often work in these businesses, exhibit a positive attitude toward their jobs due to their upbringing in the family business context (Kepner, 1983; Block et al., 2015). Additionally, the prioritization of socioemotional wealth in family businesses leads owners to maintain close, supportive relationships with their employees, considering them part of the extended family (Waterwall and Alipour, 2021; Llanos-Contreras et al., 2022a,b). Employees feeling supported and valued are more likely to experience job satisfaction and long-term commitment to the organization (Christensen-Salem et al., 2021), fostering shared values that create purpose and connection among owners, managers, and employees.

This positive working environment becomes particularly crucial when family firms face external shocks (Salvato et al., 2020; Mahto et al., 2022). During the COVID-19 pandemic, family businesses’ strong commitment to employees generated trust and loyalty, contributing to increased job satisfaction (Firfiray and Gomez-Mejia, 2021). Studies on family firms and COVID-19 highlight their provision of emotional support and resources to help employees cope with stress and uncertainty, supporting mental health and job satisfaction (Usman et al., 2021). Based on these considerations, the following hypothesis is proposed:

*H5*: Workers’ perception of involvement in a family business increases levels of job satisfaction, when facing an external shock, such as Covid-19.

As discussed earlier, family firms tend to maintain higher levels of job satisfaction due to their strong position in critical mediating factors like trust, commitment, and loyalty (Llanos-Contreras et al., 2023). However, it is essential to examine whether this pattern holds for all factors crucial in preserving job satisfaction. This study proposes that burnout and affective commitment are two critical mediating variables in this context.

Affective commitment in family firms is linked to higher job satisfaction, stemming from the emotional connection and shared purpose within the organization (Kooij et al., 2010; Swab et al., 2020). Family ties and shared values unique to family firms contribute to higher affective commitment compared to non-family firms (Vallejo, 2009; Picone et al., 2021). When employees feel emotionally invested in the organization’s survival or success during crises like the COVID-19 pandemic, they are more likely to take pride in their work, going above and beyond their duties to contribute to the company’s well-being (Lapointe and Vandenberghe, 2017). This enhanced sense of accomplishment and fulfillment can boost job satisfaction (Ahlers et al., 2017).

Family firms also create a positive work environment that promotes job satisfaction by controlling daily activities (processes) to mitigate burnout (Edmondson et al., 2019). Research suggests that work environmental conditions in family firms correlate with job satisfaction. For example, Llanos-Contreras et al. (2022b) demonstrate that organizational support, workload reduction, and minimizing job insecurity perceptions in family firms contribute to lower burnout and higher job satisfaction. When employees experience lower levels of burnout, they feel more in control of their work and personal life, leading to greater job satisfaction (Kanwar et al., 2009; Amponsah-Tawiah and Mensah, 2016; Lambrechts and Gnan, 2022). Such Human Resource Management practices are especially crucial during crises, demanding low fault tolerance rates among employees (Pinnington and Ayoko, 2021). Therefore, the analysis supports the hypotheses that both burnout and employees’ affective commitment mediate the relationship between family businesses’ work environment and job satisfaction.

*H6*a: Workers’ perception of involvement in a family business increases levels of job satisfaction through lower levels of burnout, when facing an external shock, such as Covid-19.

*H6*b: Workers’ perception of involvement in a family business increases levels of job satisfaction through Affective Commitment, when facing an external shock, such as Covid-19.

**Figure 1:**
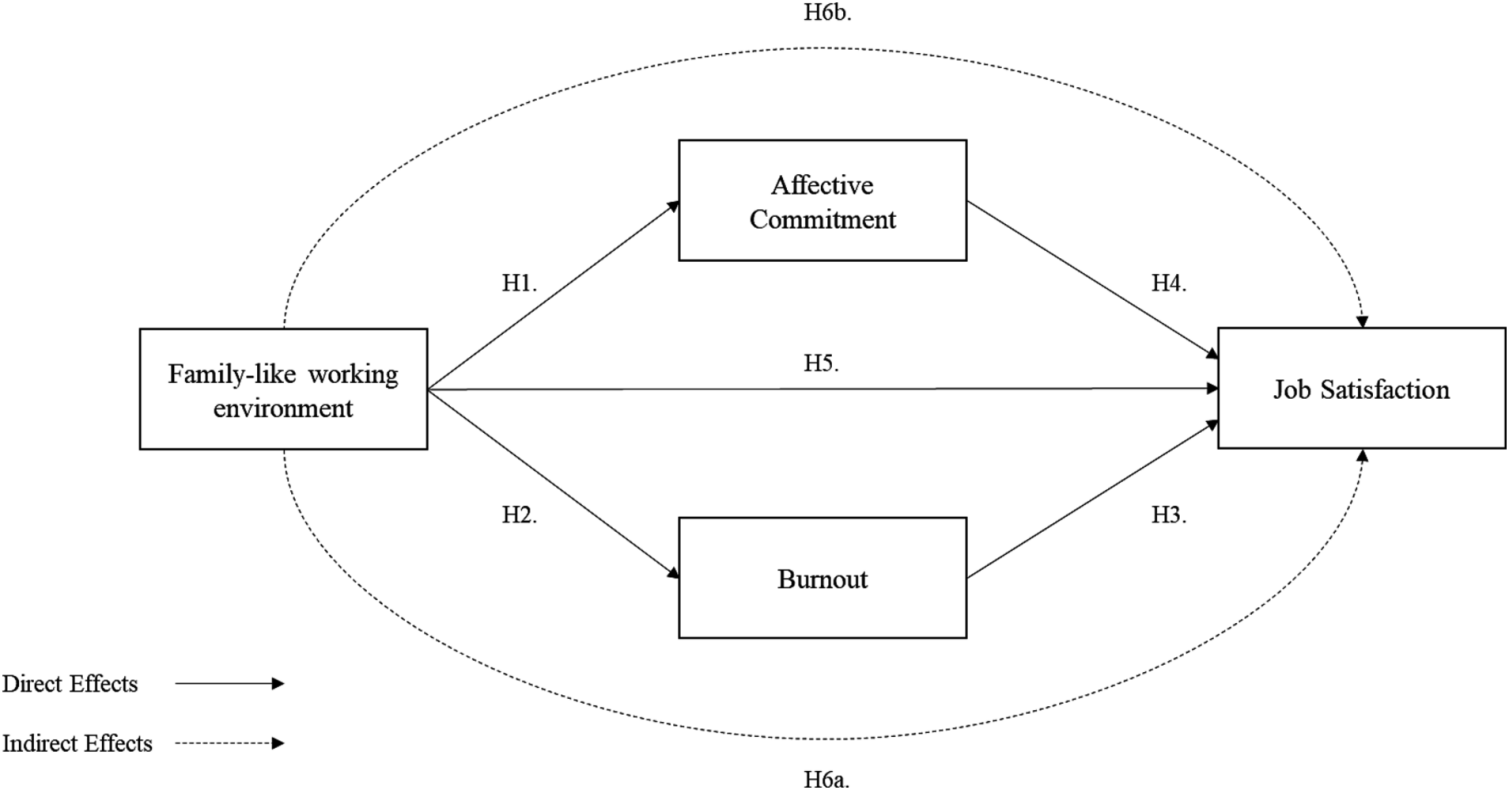
Summarizes this study’s conceptual model.

The Figure 1 summarizes the hypotheses discussed above.

## 3 Methods

Data was collected in January 2021 through an online questionnaire encompassing various domains of interest. These domains include burnout, affective commitment, family-like working environment (*family firm image*), job satisfaction, and demographic information. The survey was administered in Chile in 2021, approximately 10 months after the global declaration of the COVID-19 pandemic. A total of 516 valid questionnaires were gathered from participants with an average age of 42 years (standard deviation [SD] = 11.57). The sample consisted of 48.64% women, and most respondents held tertiary education degrees (75.58%), with 67.64% earning less than US$1,300.

The assessment of burnout levels utilized the Burnout Assessment Tool (BAT), consisting of 22 items that represent the four dimensions of burnout: exhaustion, mental distance, emotional impairment, and cognitive impairment (Sakakibara et al., 2020; Schaufeli et al., 2020). The family-like working environment (FFI) was assessed using an adapted version of the family firm image scale developed by Beck and Kenning (2015). Affective commitment (AffCom) was measured using the scale by Scrima (2015), and job satisfaction (JobSat) was evaluated through an adaptation of the scale by Eisenberger et al. (1997). In all cases, a 5-point Likert scale was used to measure the responses.

We employed a structural equation model using partial least squares (PLS-SEM) to examine the proposed hypotheses. To assess the reliability and validity of the measurement scales and the structural model, we utilized SmartPLS software (Hair et al., 2014). SEM was deemed appropriate for our study due to its ability to handle multiple relationships and integrate both observed and latent variables. PLS-SEM allows us to model relationships between observed and latent variables (measurement model) as well as relationships between latent variables (structural model), as previously noted by Hair et al. (2014), Richter et al. (2016), and Shiau et al. (2019).

## 4 Data analysis and results

### 4.1 Exploratory factor analysis

To test the psychometric properties of latent constructs an exploratory factor analysis (EFA) was implemented (Table 1). The assessment of the fit measure shows adequate levels of reliability and validity. The Cronbach’s α (>0.796) and composite reliability (CR > 0.880) confirm the internal consistency of all latent variables (Hair et al., 2014). Testing the convergent validity of the reflective models, the factor loadings (>0.700) and the average variance extracted (AVE) (>0.575) show adequate fit levels (Hair et al., 2014). Considering these results, variables were suitable for subsequent analyses.

**Table 1:** Evaluation of the measurement model.

Construct	α	Rho_A	CR	AVE	Factorial loads
FFI	0.917	0.919	0.960	0.923	>0.700^***^
Burnout	0.927	0.942	0.937	0.575	>0.700^***^
AffCom	0.796	0.809	0.880	0.709	>0.700^***^
JobSat	0.859	0.880	0.914	0.780	>0.700^***^

^***^Significance level: *ρ* < 0.01. α, Cronbach’s alpha; CR, composite reliability; AVE, average variance extracted.

To test the proposed hypotheses, a structural equations model by partial least squares (PLS-SEM) was implemented. In this study, PLS-SEM allows the modeling of the relationships between observed and latent variables (measurement model) and the relationships between latent variables (structural model) (Hair et al., 2014; Richter et al., 2016; Shiau et al., 2019).

### 4.2 Evaluation of the structural model

To assess the structural model’s relevance and predictive power, the multiple correlation coefficient (*R*^2^ = 0.505) and Stone-Geisser’s predictive relevance test (*Q*^2^ = 0.072) were used and show good fit measures (Chin and Todd, 1995; Chin, 2009). Also, the standardized root mean square residual coefficient (SRMR = 0.039) has an appropriate adjustment level (Hu and Bentler, 1998). It is possible to confirm the significance of the model, since the measures of the model’s fit show appropriate levels and predictive capacity (*R*^2^ = 0.505; *Q*^2^ = 0.072; SRMR = 0.039; Hair et al., 2017). Table 2 summarized PLS-SEM results.

**Table 2:** PLS-SEM results.

Measures	Path	ƒ^2^	*R*^2^	*Q*^2^	SRMR
*Direct effects*					
FFI → Burnout	−0.157^***^	0.025			
FFI → AffCom	0.384^***^	0.173			
FFI → JobSat	0.015^***^	0.000			
Burnout → JobSat	−0.242^***^	0.103			
AffCom → JobSat	0.581^***^	0.519			
*Specific indirect effects*					
FFI → Burnout → JobSat	0.038^***^				
FFI → AffCom → JobSat	0.223^***^				
Burnout			0.025	0.020	
Affective commitment			0.148	0.143	
Job satisfaction			0.505	0.072	
Common factor model					0.039

Bootstrapping = 5,000. Significance level: ^*^ρ < 0.10; ^**^ρ < 0.05; ^***^ρ < 0.01.

**Figure 2:**
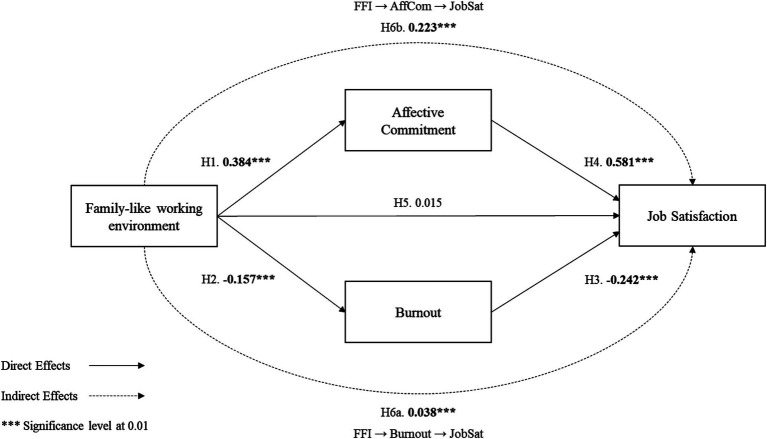
Hypotheses testing.

Findings suggest that workers’ perception of involvement in a family business enhances the sense of employees’ affective commitment toward the firm, i.e., *Hypothesis 1* is supported. *Hypotheses 2* are accepted; as, workers’ perception of involvement in a family business decreases burnout levels. High levels of worker burnout decrease job satisfaction, supporting *Hypothesis 3*. Also, a higher level of affective commitment increases workers’ job satisfaction, supporting *Hypothesis 4*. Regarding *Hypothesis 5*, workers’ perception of involvement in a family business shows non-significant effects on job satisfaction, i.e., *Hypothesis 5* is rejected. The specific indirect effects show that employees’ burnout levels and affective commitment moderate the relationship workers’ perception of involvement in a family business and job satisfaction. Workers’ perception of involvement in a family business has positive effects on job satisfaction even when high levels of burnout are observed. Also, relationship between workers’ perception of involvement in a family business and job satisfaction is positive through affective commitment. Therefore, these results support *Hypothesis 6* (a and b). Figure 2 summarizes the hypotheses testing results.

## 5 Discussion

This study aims to investigate the impact of workers’ perception of involvement in a family business on affective commitment, burnout levels, and job satisfaction during external shocks, such as the COVID-19 pandemic. Additionally, it explores the indirect influence of workers’ perception of involvement in a family business on job satisfaction through the mediating factors of burnout and affective commitment. Employing Partial Least Squares Structural Equation Modelling (PLS-SEM), the results supported all proposed hypotheses except for H5. Family firms are recognized for their ability to face external shocks and unexpected events successfully (Llanos-Contreras et al., 2020, 2023). However, previous research has not provided conclusive evidence regarding these firms’ ability to develop a working environment that promotes positive occupational health and employee job satisfaction. For instance, these firms tend to offer lower salaries and fewer prospects for career advancement to non-family employees, as indicated by Block et al. (2018) and Waterwall and Alipour (2021). On the other hand, they are often perceived as responsible caretakers who ensure significant job stability and foster social connections, as noted by Arregle et al. (2007), Hauswald et al. (2016), and Llanos-Contreras et al. (2022a,b). In this context, by comprehending the mechanisms employed by family firms to endure exogenous events, such as the COVID-19 pandemic, this study identifies unique attributes and mechanisms that enable them to navigate external disruptions effectively, thereby preserving the occupational health and job satisfaction of their employees.

Our findings suggest that a family firm’s working environment aligns with higher levels of employees’ affective commitment when an organization faces an external disruption, such as the COVID-19 pandemic. This is in line with previous research indicating that family firms develop a more robust organizational climate (O’Regan and Quigley, 1993; Tabor et al., 2018), which is acknowledged as an important driver of employees’ commitment. Family businesses promote a family-like work environment as part of their organizational culture, values, traditions, and identity (Miller and Le Breton-Miller, 2005; Cennamo et al., 2012; Llanos-Contreras et al., 2022b). Our findings shed additional light on these ideas. Preserving a stronger affective commitment toward the firm is considered an important driver of the resilience advantage of family firms in successfully facing external disruptions. This has been widely documented in previous research (Bassanini et al., 2013; Llanos-Contreras et al., 2019, 2023; Bartik et al., 2020; Marshall and Schrank, 2020; Christensen-Salem et al., 2021; Mahto et al., 2022). This research shows that workers’ perception of involvement in a family business increases the worker’s affective commitment during an external shock. Consistent with previous research (Llanos-Contreras et al., 2019; Bartik et al., 2020), our study suggests that family business workers feel a higher sense of belonging and loyalty, which helps keep emotions, psychological risk, and employee satisfaction under control when disruptions are faced. Therefore, it may suggest that a family-like working environment provides conditions that help employees efficiently manage potential psychological risks when facing uncertain scenarios.

It is expected that employees’ burnout would increase in working conditions of work overload, risky work environments, and a mismatch between the job description and the actual job conditions, among other stress drivers’ scenarios (Burke, 1989; Savicki and Cooley, 1994; Swendiman et al., 2019; Torrès et al., 2021; Lyu et al., 2023). During the COVID-19 pandemic, these conditions arose more frequently in employees across all types of firms in different economic sectors (Prado-Gascó et al., 2020). Consequently, many people suffered a detriment to their mental health (Apedo-Amah et al., 2020). Our findings suggest that family firms managed these elements more efficiently, resulting in more controlled levels of burnout. This is in line with previous studies suggesting that family firms have advantages in managing external disruptions, maintaining a resilient workplace, and offering job security in times of crisis (Alonso-Dos-Santos and Llanos-Contreras, 2019; Llanos-Contreras et al., 2020). In this same line, this study is also in line with research suggesting that the worker feeling part of a family firm decreases burnout levels, even when an external shock happens (Cruz et al., 2012; Fotiadis et al., 2019; Kraus et al., 2020; Schwaiger et al., 2022). Thus, the premise that a family firm’s work environment offers a social context in which employees perceive protective effects, as proposed by Rauscher et al. (2012), is supported and maintained during external disruptions. Moreover, it may be suggested that these firms have a superior capability to manage organizational routines through crises generated from external disruption. Consequently, family firms efficiently manage crises to maintain a supportive work environment, which is recognized by employees and may represent a resilience advantage for these businesses (O’Regan and Quigley, 1993; Bassanini et al., 2013; Tabor et al., 2018; Christensen-Salem et al., 2021).

This study also provides further support to previous research suggesting that affective commitment has a positive influence on job satisfaction, while burnout has a negative influence on this variable. Regarding the relationship between affective commitment and job satisfaction, it is known that high levels of affective commitment enhance job satisfaction. Moreover, in times of crisis, workers’ affective commitment tends to increase, strengthening family firms’ capacity to cope with adverse situations (Cruz et al., 2012; Koo et al., 2020; Chanana, 2021). Similarly, the results also confirm the negative relationship between worker burnout and job satisfaction in previous research, highlighting the influence of mental health on job satisfaction and employee well-being (Edmondson et al., 2019; Madigan and Kim, 2021; Lea et al., 2022; Anand et al., 2023). This suggestion is prevalent in external disruption scenarios (Ninaus et al., 2021; Kraus et al., 2023). While this finding may not be novel, confirming the role played by these two variables in efficiently managing the stress generated by external disruptions, such as the COVID-19 pandemic, is crucial. Building upon the aforementioned research, we propose that family firms possess a stock of commitment from employees (affective commitment), providing them with an advantage to face adverse scenarios and preserve higher levels of job satisfaction. Similarly, the results of this study confirm that family firms have a superior ability to deal with burnout when facing unexpected disruptions. Drawing on Llanos-Contreras et al. (2020) and Mahto et al. (2022) we suggest that such ability is rooted in their prioritization of preserving socioemotional wealth, including commitment to continuity and social ties with employees, among other factors. This orientation leads them to more efficiently manage organizational routines (which, as discussed above, are central in keeping burnout under control) through crises, resulting in better levels of job satisfaction. This finding aligns with Christensen-Salem et al. (2021), who argue that pursuing socioemotional wealth explains why family businesses adopt more caring practices toward employees, despite potentially providing fewer or less comprehensive formal Human Resource Management (HRM) programs and benefits.

Finally, the study also provides support for the idea that a family-like working environment positively influences job satisfaction. This is significant because it suggests that in times of crises, family firms are capable of leveraging their distinctive family-like working environment to impart stability and security to employees (Alonso-Dos-Santos and Llanos-Contreras, 2019; Llanos-Contreras et al., 2020; Madden et al., 2020; Le Breton-Miller and Miller, 2022). Importantly, the results do not provide support for the expected positive direct influence of the family firm’s working environment on job satisfaction. However, they do confirm the suggested indirect influences through affective commitment and burnout. This may imply that the family firm condition alone may not be sufficient to increase job satisfaction. However, a family-like working environment would create conditions, such as enhanced affective commitment and more controlled burnout, through which job satisfaction can be preserved when these organizations face disruptive scenarios. Therefore, it is suggested that the structural conditions (in terms of affective commitment) and organizational dynamics (which allow keeping burnout under control) implemented by family firms during the pandemic are effective and valued by the employees.

## 6 Contributions to theory and practice

This study makes significant contributions to both theory and practice, which will be discussed below. Firstly, it adds to the body of literature examining how family businesses dealt with challenges posed by external disruptions, such as the COVID-19 pandemic (Kraus et al., 2020; Llanos-Contreras et al., 2023). This research sheds light on the critical role played by employees’ affective commitment and the control of burnout in preserving job satisfaction when firms face disruptive scenarios. It also provides insight into these firms’ superior ability to cope with uncertainty and stress driven by an external shock. For family firms, continuity and preserving the family legacy are ultimate goals (Kotlar and De Massis, 2013). The contribution made regarding how these firms manage crises generated from external disruptions is central to understanding the resources and capabilities that support the longevity of these companies. In relation to specific research on COVID-19 and family firms, earlier studies provide insights into these firms’ responses to the upheavals brought about by the pandemic from a broad management perspective. In contrast, our research specifically concentrated on the commitment and management of human resources, recognizing it as a crucial factor influencing these firms’ capacity to navigate this abrupt external disruption.

A second important contribution to theory is to the literature on family business resilience (Danes et al., 2009; Salvato et al., 2020). Previous research has provided insights into these firms’ ability to manage organizational decline and sustain their business (Llanos-Contreras and Jabri, 2019). Other studies have shown strategies and mechanisms developed by family firms to navigate financial distress and minimal financial rewards (Glover and Reay, 2015). More recently, research has informed on the mechanisms, strategic logic, and motivations behind a family firm’s strategic evolution to support sustainable longevity (Welsh et al., 2023). Our study suggests that job satisfaction is critical for these firms’ resilience and continuity. It indicates that merely being a family-owned business does not automatically ensure increased job satisfaction when the organization encounters an external disruption and needs to be resilient and adapt to a new context. This outcome is achievable only when both employees’ affective commitment and effectively managed (reduced) burnout are in place.

Ultimately, this research adds value to the exploration of human resources management in family businesses (Lambrechts and Gnan, 2022; Pelaez-Leon and Sanchez-Marin, 2023) by pinpointing and examining specific resources that confer advantages to these firms, such as employee affective commitment. Additionally, the study verifies their capability to maintain lower levels of burnout compared to non-family firms when confronted with external disruptions. A recent work by Christensen-Salem et al. (2021) addresses the debate on whether family firms offer better or worse work environments than non-family firms. Drawing from socioemotional wealth theory and the behavioral agency model, they argue that family owners aim to enhance organizational caring perceptions among employees. Our study suggests that such perceptual conditions would provide an important advantage when family firms face stressful scenarios caused by external shocks. Similarly, we found that under a scenario of external shock, family firms have a superior ability to manage burnout. Recent research from Peláez-León and Sánchez-Marín (2022) indicates that family firms implement High-Performance Human Resources Systems (HPWS) as a mechanism to preserve their socioemotional wealth (SEW). We suggest that our finding regarding the efficient way family firms manage crises and control employees’ burnout is also a mechanism to preserve socioemotional wealth. It informs on the critical role played by human resource practices not only in preserving job satisfaction but also in maintaining good levels of performance when organizations face adverse scenarios.

From a practical perspective, the study’s results emphasize the critical role of employees’ affective commitment and the control of burnout for family business managers in navigating challenges posed by external disruptions and contributing to the overall resilience and longevity of their organizations. Recognizing the relevance of employees’ satisfaction during episodes of significant disruption is crucial for family business managers, and job satisfaction is identified as a key factor contributing to the resilience of family businesses. Managers should regularly assess and address factors influencing job satisfaction to maintain a positive work environment. Leveraging a robust position in controlling burnout and fostering employee affective commitment enables family business owners and managers to not only sustain higher levels of job satisfaction but also capitalize on business opportunities arising during external disruptions. This structural advantage positions family firms to face adversities more effectively, potentially improving their competitive position when industries confront environmental threats, competitive difficulties, and external challenges. Conversely, non-family firms can adopt strategies from family firms to preserve job satisfaction during external shocks and cultivate resilience capabilities. Creating a family-like working environment that reinforces workers’ affective commitment toward the firm may prove effective for both family and non-family firms in successfully weathering external shocks. Recognizing the positive impact of a family-like environment on job satisfaction, even in the face of high-stress levels generated by events like the COVID-19 pandemic, is crucial. Both family and non-family firms can mitigate the adverse effects of external disruptions on workers’ mental health, thereby safeguarding overall business performance.

## 7 Conclusion

In conclusion, this study conducted during the COVID-19 pandemic sheds light on the resilience of family businesses in Latin America, particularly in Chile. The findings indicate that employees in family firms experience higher levels of job satisfaction when confronted with the challenges of the pandemic. This observation suggests that the working environment fostered by family firms in the sample exhibits greater resilience to unexpected external shocks. Consequently, it is inferred that family firms establish a distinctive and robust organizational climate, proving particularly advantageous in facing adversities arising from external disruptions. Crucially, the study reveals that this advantage is achieved through affective commitment and controlled burnout as mediating variables. Theoretical implications propose that these variables play a crucial role in enabling these firms to navigate external disruptions. Affective commitment is regarded as a resource advantage, representing a foundational condition that offers a structural edge challenging for non-family firms to replicate. On the other hand, the family firms’ capacity to manage burnout is seen as rooted in their proficiency in handling organizational processes, particularly in stressful scenarios, as suggested by prior research. This underscores the importance of resource asymmetries and organizational process management at the occupational health level in preserving employee satisfaction under challenging circumstances.

### 7.1 Limitations and future research

Workers’ perceptions of the severity of disruptive events can influence their evaluation of organizational support, and future studies could explore how such perceptions affect preferences for family-like working environments. Additionally, the study did not consider new workplace configurations like remote work implemented during the COVID-19 pandemic. An interesting avenue for research is investigating how remote work influences the benefits of a family-like environment during a pandemic. Changes in employment contracts, such as salary reductions, may also impact perceptions of organizational support and job satisfaction. This raises questions about how evolving workplace dynamics and contractual changes interact with the advantages of a family-like working environment in sustaining employee well-being during challenging circumstances. Exploring these aspects would contribute to a more comprehensive understanding of the dynamics between disruptive events, work configurations, and organizational support.

## Data availability statement

The raw data supporting the conclusions of this article will be made available by the authors, without undue reservation.

## Ethics statement

Ethical review and approval was not required for the study on human participants in accordance with the local legislation and institutional requirements. The participants provided their written informed consent to participate in this study. Written informed consent was obtained from the individual(s) for the publication of any potentially identifiable images or data included in this article.

## Author contributions

MI: Conceptualization, Data curation, Formal analysis, Funding acquisition, Investigation, Methodology, Project administration, Resources, Software, Supervision, Validation, Visualization, Writing – original draft, Writing – review & editing. NA-V: Conceptualization, Data curation, Formal analysis, Funding acquisition, Investigation, Methodology, Project administration, Resources, Software, Supervision, Validation, Visualization, Writing – original draft, Writing – review & editing. OL-C: Conceptualization, Data curation, Formal analysis, Funding acquisition, Investigation, Methodology, Project administration, Resources, Software, Supervision, Validation, Visualization, Writing – original draft, Writing – review & editing.
